# MicroRNA‐92b‐5p modulates melatonin‐mediated osteogenic differentiation of bone marrow mesenchymal stem cells by targeting ICAM‐1

**DOI:** 10.1111/jcmm.14490

**Published:** 2019-07-14

**Authors:** Yuan Li, Chao Feng, Manqi Gao, Mengyu Jin, Tianyi Liu, Ye Yuan, Gege Yan, Rui Gong, Yi Sun, Mingyu He, Yutuo Fu, Lai Zhang, Qi Huang, Fengzhi Ding, Wenya Ma, Zhenggang Bi, Chaoqian Xu, Natalia Sukhareva, Djibril Bamba, Russel Reiters, Fan Yang, Benzhi Cai, Lei Yang

**Affiliations:** ^1^ Department of Pharmacology (The State‐Province Key Laboratories of Biomedicine‐Pharmaceutics of China, Key Laboratory of Cardiovascular Research, Ministry of Education) College of Pharmacy, Harbin Medical University Harbin China; ^2^ College of Pharmacy University of Cincinnati Cincinnati Ohio; ^3^ Department of Pharmacy The Second Affiliated Hospital of Harbin Medical University Harbin China; ^4^ Department of Orthopedics The Second Affiliated Hospital of Harbin Medical University Harbin China; ^5^ Department of Orthopedics The First Affiliated Hospital, Harbin Medical University Harbin China; ^6^ Department of Cellular and Structural Biology The University of Texas Health Science Center at San Antonio San Antonio Texas

**Keywords:** BMSCs, ICAM‐1, melatonin, MiRNA, osteogenic differentiation, osteoporosis

## Abstract

Osteoporosis is closely associated with the dysfunction of bone metabolism, which is caused by the imbalance between new bone formation and bone resorption. Osteogenic differentiation plays a vital role in maintaining the balance of bone microenvironment. The present study investigated whether melatonin participated in the osteogenic commitment of bone marrow mesenchymal stem cells (BMSCs) and further explored its underlying mechanisms. Our data showed that melatonin exhibited the capacity of regulating osteogenic differentiation of BMSCs, which was blocked by its membrane receptor inhibitor luzindole. Further study demonstrated that the expression of miR‐92b‐5p was up‐regulated in BMSCs after administration of melatonin, and transfection of miR‐92b‐5p accelerated osteogenesis of BMSCs. In contrast, silence of miR‐92b‐5p inhibited the osteogenesis of BMSCs. The increase in osteoblast differentiation of BMSCs caused by melatonin was attenuated by miR‐92b‐5p AMO as well. Luciferase reporter assay, real‐time qPCR analysis and western blot analysis confirmed that miR‐92b‐5p was involved in osteogenesis by directly targeting intracellular adhesion molecule‐1 (ICAM‐1). Melatonin improved the expression of miR‐92b‐5p, which could regulate the differentiation of BMSCs into osteoblasts by targeting ICAM‐1. This study provided novel methods for treating osteoporosis.

## 1 INTRODUCTION

Millions of humans in the ageing populations, especially post‐menopausal women suffer from osteoporosis throughout the world.^1^ Osteoporosis is a progressive systemic skeletal disease which is characterized by low bone mass and microarchitecture deterioration of bone tissue, leading to reduced bone mineral density (BMD) and elevated risk of fractures.^2^ Osteoporosis is attributed to the dysfunction of bone metabolism, which is caused by the imbalance between new bone formation by osteoblasts and old bone resorption by osteoclasts. Osteoblasts in the bone environment are mainly derived from bone marrow mesenchymal stem cells (BMSCs) which have the capacity to differentiate into various cell types, including osteoblasts, chondrocytes and adipocytes.^3^, ^4^ Thus, BMSCs, with inherent osteogenic differentiation potential, have been widely used for cell therapy and tissue regeneration based on their multi‐lineage differentiation potential and easy access.^5^ The dysfunction of BMSCs because of pathological stimuli or gene mutations is also involved in the occurrence and development of osteoporosis. Some studies uncovered that BMSCs engraftment may promote osteogenesis and produce therapeutic effects on bone fractures in animals.^6^, ^7^ Thus, how to effectively promote osteogenic differentiation of BMSCs has been regarded as an important strategy for osteoporosis treatment.

Melatonin (N‐acetyl‐5‐methoxytryptamine) is a neurohormone produced and secreted by the pineal gland, and pineal synthesis is primarily regulated by the light and dark environment working via suprachiasmatic nucleus.^8^ Melatonin plays a critical role in a wide variety of physiological functions, such as circadian rhythms, renal function, hormone secretion, antioxidant defence, immune responses, reproduction control, lipid metabolism, tooth development, anti‐tumour capacity and etc.^9^, ^10^, ^11^, ^12^ In addition, accumulating evidence has revealed that melatonin is also involved in various diseases, including periodontal disease, cancer, diabetes, pulmonary fibrosis, spinal cord injury, Alzheimer's disease and hepatic fibrosis.^13^, ^14^ Recently, the association between melatonin and bone development has received increased attentions. For example, melatonin accelerates osteoblast differentiation of pre‐osteoblastic MC3T3 cell lines and improves the bone‐forming capacity under hypoxic condition.^15^ Furthermore, treatment with melatonin stimulates matrix mineralization and promotes new bone formation of mice.^16^ Our previous study suggested that melatonin may reverse iron overload‐induced dysfunction of BMSCs.^17^ However, little is known regarding the mechanisms of melatonin promoting the osteogenic differentiation of BMSCs, which need further investigations.

MicroRNAs (miRNAs) are a family of abundant, endogenously expressed, double‐stranded small non‐coding RNAs of about 20‐25 nucleotides.^18^, ^19^ MiRNAs function as key regulators of gene expression by binding to the 3'‐UTR of their target mRNAs and play a critical role in a wide variety of biological processes, including cell proliferation, cell apoptosis, cell fate and organ development.^20^, ^21^ In addition, miRNAs participate in multiple diseases, such as cancers, diabetes mellitus, heart failure, liver injury, cerebrovascular disease and glaucoma.^22^, ^23^, ^24^ Furthermore, recent studies have reported that miRNAs are related to bone metabolism and bone regeneration. Research has shown that 10‐11 translocation (Tet) plays a key role in maintaining BMSCs and bone homeostasis by controlling miR‐297a‐5p, miR‐297b‐5p, and miR‐297c‐5p release.^25^ Besides, many studies have revealed that miRNAs play an important role in the melatonin‐mediated biological functions.^26^, ^27^, ^28^, ^29^ For example, it has been reported that melatonin successfully accelerates the chondrogenic by increasing the expression of miR‐526b‐3p and miR‐590‐5p by regulating SMAD7.^30^ Nevertheless, the information about the roles of miRNAs in the protection of melatonin on osteogenesis of BMSCs has not been fully explored.^9^

Herein, we demonstrated that melatonin has a role in osteogenesis of BMSCs, and melatonin regulates osteoblast differentiation by increasing the expression of miR‐92b‐5p which directly targets intracellular adhesion molecule‐1 (ICAM‐1). The findings suggest that melatonin may be an effective therapeutic treatment for osteoporosis.

## 2 MATERIALS AND METHODS

### 2.1 BMSC isolation and culture

All animal procedures were performed in accordance with the Animal Experimental Ethics Committee of Harbin Medical University, Harbin City, China. Sex‐, age‐ and weight‐matched C57BL/6J mice (female, 8 weeks old, 16‐18 g) were obtained from the Experimental Animal Center of Second Affiliated Hospital of Harbin Medical University. BMSCs were isolated according to the procedure as described in our previous studies.^31^, ^32^, ^33^ Briefly, the animals were intraperitoneally injected with pentobarbital sodium (Sigma, USA) and anaesthetized, and then killed. The femur and tibias were separated and BMSCs were flushed from bone marrow cavity using 4 mL volume of normal culture medium (Cyagen, USA). And these cells were regarded as passage 0 (P0). BMSCs were cultured and maintained in 25 cm^2^ culture flasks and maintained in a cell culture incubator containing 5% CO_2_ and 95% humidity at 37°C (Thermo, USA).

### 2.2 Transfection

To identify the role of miRNAs in osteogenic differentiation of BMSCs, miR‐92b‐5p mimics and miR‐92b‐5p AMO were designed and synthesized by GenePharma, China. Their corresponding negative controls (NC) were also provided by the manufacturer. The sequences of miR‐92b‐5p mimics were: primary chain, 5'‐AGGGACGGGACGUGGUGCAGUGUU‐3', and passenger chain, 5'‐CACUGCACCACGUCCCGUCCCUUU‐3'. The sequence of miR‐92b‐5p AMO was 5'‐AACACUGCACCACGUCCCGUCCCU‐3'. The concentrations of miR‐92b‐5p mimics and miR‐92b‐5p AMO used were 50 and 100 nmol/L respectively. When the cells grew to 50%‐60%, BMSCs were transfected with miRNAs in the presence of X‐treme (Roche, Switzerland) and Opti‐MEM Reduced Serum Medium (Invitrogen, USA) according to the manufacturer's protocols.

### 2.3 Osteogenesis induction

To induce osteogenic differentiation of BMSCs, the cells were cultured in 6‐well or 24‐well plates under standard culture conditions of 37°C and 5% CO_2_. The cells were maintained in normal culture medium (Cyagen, USA) until 80% confluence and then they were differentiated into osteoblasts using osteogenic differentiation‐inducing medium (Cyagen, USA). Osteogenic differentiation‐inducing medium was composed of 175 mL culture medium, 10% FBS, 1% glutamine, 1% penicillin‐streptomycin, 0.2% ascorbic acid, 1% β‐glycerophosphate and 0.01% dexamethasone. The cells were induced into osteoblasts for 14 days and the medium was changed every 3 days.

### 2.4 Melatonin treatment

Melatonin was purchased from Sigma (USA) and 23.228 mg melatonin was totally dissolved in 10 mL double distilled water. The concentration of melatonin at 10 mmol/L was regarded as the working solution used for further experiments. For the analysis evaluating the role of melatonin, the cells were grown in the presence of differentiation‐inducing medium combined with different concentration of melatonin for 14 days. The cells without melatonin treatment were regarded as the controls.

### 2.5 Alizarin red S staining

To examine the activity of osteoblasts, the BMSCs were placed in 24‐well plates and were induced into osteoblasts. Mineralization of cells was assessed on day 14 by performing alizarin red S (ARS) staining and the cells were washed with PBS gently three times. Then the cells were embedded in 4% PFA (Solarbio, China) for 30 minutes at room temperature. Then the cells were incubated using 1 mL ARS staining solution which was purchased from Cyagen (USA) for 30 minutes at room temperature. ARS staining solution chelated with calcium of cells and then formed an alizarin red‐calcium complex which exhibited a bright red colour. After three washes with PBS to remove needless unbound stains, the degree of osteogenic differentiation was observed under an optimal microscope (Nikon, Japan). The experiment was performed at least three times.

### 2.6 Alkaline phosphatase staining

To assess the calcium deposition by osteoblasts, BMSCs were cultured in osteogenic differentiation‐inducing medium for 14 days. After osteogenic differentiation, the cells were stained with alkaline phosphatase (ALP) staining solution for 4 hours at 37°C using published protocol. The ALP staining solution was a mixture of 3% β‐glycerophosphate disodium salt hydrate (Sigma, USA), 2% sodium pentobarbital (Peking Tongxian Yucai fine chemical, China), 2% calcium chloride (Tianjin Bodi Chemical, China) and 2% magnesium sulphate (Tianjin Haijing Fine Chemical, China). The cells were incubated with ALP staining solution for 4 hours at 37°C. After incubation four 4 hours, 2% cobalt nitrate was quickly added to the plates for 5 minutes and the cells were rinsed three times with PBS. Then the cells were incubated in 0.2% ammonium chloride solution for 1 minute. Thereafter, 10 pictures were randomly taken and calcium‐rich deposits were observed under a microscope (Nikon, Japan). The gradation of colour represented the intensity of calcification of cells.

### 2.7 RNA isolation, cDNA synthesis and real‐time qPCR

To evaluate the expression level of osteogenic marker genes and miR‐92b‐5p, we performed real‐time qPCR analysis according to the protocol. In brief, total RNA was extracted from BMSCs after treatment with melatonin or miRNAs by using TRIzol reagent (Invitrogen, USA). cDNA was converted from 500 ng of total RNA using reverse transcription cDNA synthesis kit (Vazyme, China) according to the manufacturer's protocol. Then, 1 μL of synthesized cDNA, 10 μL of SYBR Green Master Mix (Vazyme, China) and 1 μL of specific primers were used for amplification. Subsequently, real‐time qPCR was performed on a Roche 480 real‐time PCR System with the following conditions: 95°C for 30 seconds followed by 40 cycles at 95°C for 5 seconds and 60°C for 30 seconds. For the expression of the osteogenic‐related genes including ALP, Osterix (Sp7), bone morphogenetic protein 2 (BMP2), bone morphogenetic protein 4 (BMP4), runt‐related transcription factor (Runx2), collagen type αI (Collagen‐1), osteocalcin (OCN) and osteopontin (OPN), the expression of glyceraldehyde 3‐phosphate dehydrogenase (GAPDH) gene was used as an internal control for each sample. For the expression of miR‐92b‐5p, U6 was used as a housekeeping gene for standardization. The relative expression of these genes was quantified by the 2^−△△CT^ method. The sequences of specific‐gene primers used for real‐time qPCR are listed in detail in Table 1.

**Table 1:** The sequences of primers

Target genes		Primer sequences
ALP	Forward	ACAACCTGACTGACCCTTCG
	Reverse	TCATGATGTCCGTGGTCAAT
Collagen‐1	Forward	CAGCCGCTTCACCTACAGC
	Reverse	TTTTGTATTCAATCACTGTCTTGCC
BMP2	Forward	CCTTGCTGACCACCTGAACT
	Reverse	AACATGGAGATTGCGCTGA
BMP4	Forward	TCGTTACCTCAAGGGAGTGG
	Reverse	ATGCTTGGGACTACGTTTGG
Runx2	Forward	AGAAGGCACAGACAGAAGCTTGA
	Reverse	AGGAATGCGCCCTAAATCACT
Sp7	Forward	AGAGGTTCACTCGCTCTGACGA
	Reverse	TTGCTCAAGTGGTCGCTTCTG
OCN	Forward	TTCTGCTCACTCTGCTGACC
	Reverse	TTTGTAGGCGGTCTTCAAGC
OPN	Forward	ACACTTTCACTCCAATCGTCC
	Reverse	TGCCCTTTCCGTTGTTGTCC
GAPDH	Forward	CATCACTGCCACCCAGAAGAC
	Reverse	CCAGTGAGCTTCCCGTTCAG
U6	Forward	GCTTCGGCAGCACATATACTAAAAT
	Reverse	CGCTTCACGAATTTGCGTGTCAT
	RT	CGCTTCACGAATTTGCGTGTCAT
mmu‐miR‐92b‐5p	Forward	GCCGAGGGACGGGACGTGGTG
	Reverse	CAGCCACAAAAGAGCACAAT
	RT	CCTGTTGTCTCCAGCCACAAAAGAGCACAATATTTCAGGAGACAACAGG
ICAM‐1	Forward	GGAAGGGAGCCAAGTAACTGTGAAG
	Reverse	GAGCGGCAGAGCAAAAGAAGC

### 2.8 Western blot

Western blot was conducted to examine the protein expression of target genes, including ICAM‐1. Briefly, BCA protein assay kit (Beyotime, China) was used to quantify the protein samples. BMSCs were grown in 6‐well plates at an initial density of 1 × 10^5^ cells/cm^2^ and transfected with miRNAs. Then, after osteoblastic differentiation induced by OM for additional 14 days, the cells were harvested. Cell pellets were obtained after centrifugation at 15000 rpm, washed with PBS and mixed with ice‐cold lysis buffer as described in a previous study.^34^, ^35^ Then cellular extracts were shifted to SDS‐PAGE and transferred to a PVDF membrane (Millipore, USA). The membranes were blocked with 5% non‐fat milk with gentle shaking for 2 hours. After washing with TBST for three times, the membranes were incubated with specific primary antibodies overnight with a gentle shaking at 4°C. Subsequently, the membranes were incubated with the secondary antibody for 1 hour at room temperature. After washing, strength of each membrane was detected by an ImageJ software (National Institutes of Health, USA) using Odyssey Infrared Imager. Anti‐ICAM‐1 (CST, USA) was purchased from Omnimabs. The expression level of β‐actin was used as the reference.

### 2.9 Establishment of OVX‐induced osteoporotic mice

To analyse the potential of osteogenesis of BMSCs in osteoporotic mice, 8‐week‐old female C57BL/6J mice were purchased to construct an ovariectomy‐induced osteoporotic mouse model based on established protocol.^36^ All the work was performed in compliance with the guidelines of the National Institutes of Health Guidelines for the Care and Use of Laboratory Animals. The mice were randomly divided into Sham group or OVX group. After an intraperitoneal injection of pentobarbital sodium, the mice underwent bilateral ovariectomy (OVX) or sham operation (Sham) respectively. Successful OVX models were defined as osteoporotic mice in contrast to the Sham group. After additional 4 weeks of feeding, the mice were killed and BMSCs were isolated from tibias and femurs as mentioned above. The cells from the two groups were cultured for osteogenic potential analysis.

### 2.10 Immunofluorescence staining

To identify the osteoblast activity in response to miRNA treatments, the protein expression of Runx2 was measured by immunofluorescence staining. In short, BMSCs were seeded in glass slides at a density of 2 × 10^4^ cells/cm^2^ and transfected with miR‐92b‐5p mimics, miR‐92b‐5p AMO and their corresponding negative controls respectively. After exposure to OM for 4 days, differentiated cells were harvested for further experiment. After fixation by 4% PFA for 30 minutes and blocking with 5% BSA for 1 hour at room temperature, the cells were incubated with anti‐Runx2 (Abcam, UK) on a shaker at low speed at 4°C overnight. After rinsing with PBS, the cells were incubated in the secondary antibody against the species of primary antibody for 1 hour at room temperature. Then, DAPI solution was applied to counterstain the nucleus of cells followed three more rinses with PBS in a dark room. Finally, photographs were captured under a fluorescence microscope (Olympus, Japan).

### 2.11 Luciferase reporter assay

The candidate targets of miR‐92b‐5p were predicted by TargetScan (http://www.targetscan.org). Among these target genes, we chose ICAM‐1 for further study. MiRNA‐related database TargetScan Human 5.1 and BiBiServ were applied to predict the binding sequences between miR‐92b‐5p and ICAM‐1. To visualize whether miR‐92b‐5p regulated the mRNA expression of ICAM‐1, luciferase reporter assay was performed. The wild‐type (WT) 3'‐UTR or the mutant (mut) 3'‐UTR of ICAM‐1 was amplified and cloned into psi‐CHECK2 plasmids. Then, the WT 3'‐UTR or mut 3'‐UTR of ICAM‐1, miR‐92b‐5p mimics or miR‐92b‐5p AMO were co‐transfected into HEK293T cells using Lipofectamine™ 2000 (Invitrogen, USA) and Opti‐MEM medium. After 48 hours, the cells were harvested and analysed for luciferase activity by Dual‐Luciferase Reporter Assay System (Promega, USA).

### 2.12 Statistical analysis

All experiments were repeated three times independently. All statistical analysis was performed by GraphPad Prism 5.0 software (GraphPad Software, USA). *P* < 0.05 was regarded statistically significant.

## 3 RESULTS

### 3.1 Melatonin promotes the osteogenic differentiation of BMSCs

**Figure 1:**
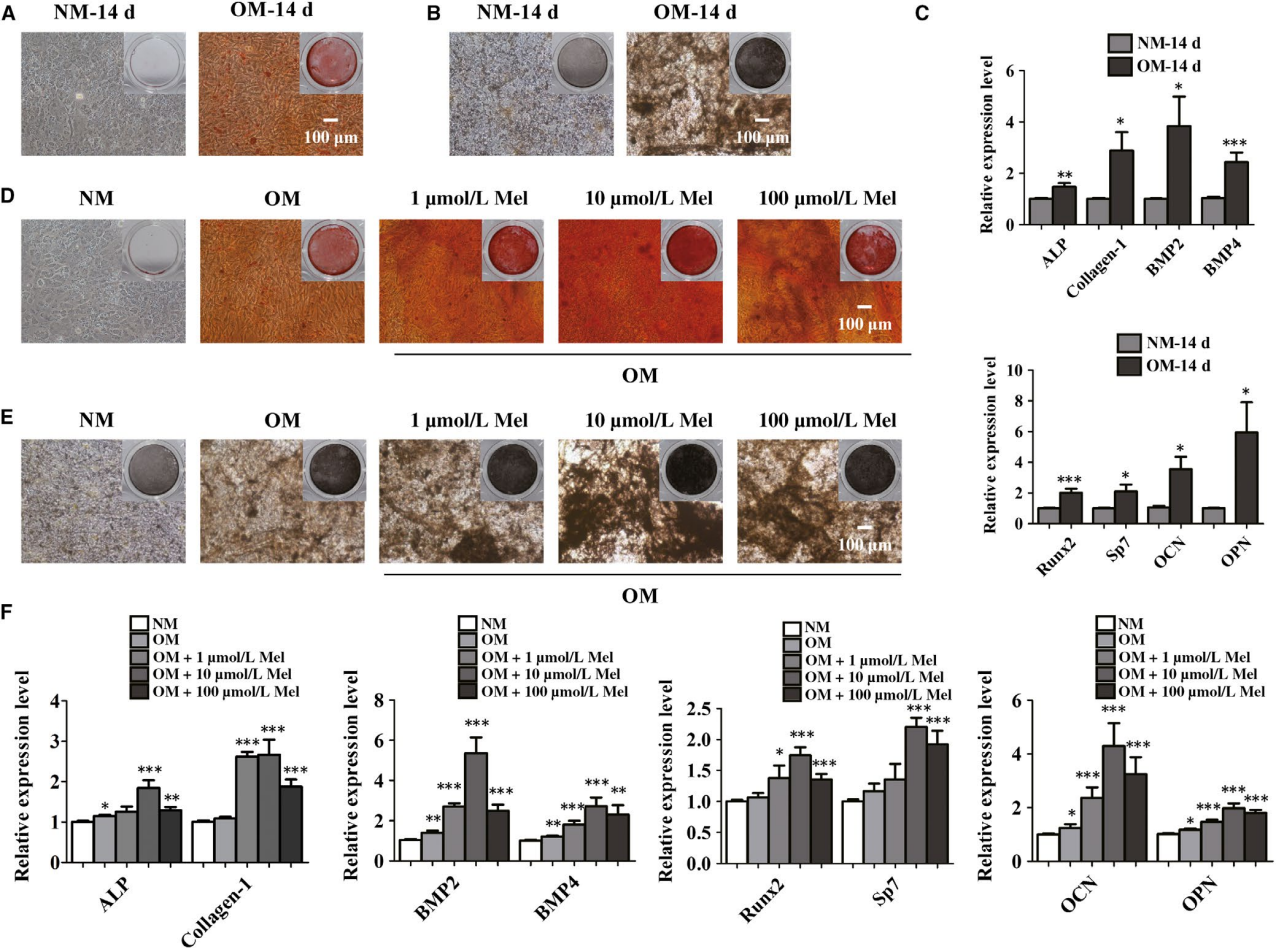
The role of melatonin in the osteogenic differentiation of bone marrow mesenchymal stem cells (BMSCs). (A and B) alizarin red S staining (ARS) (A) and alkaline phosphatase (ALP) (B) staining of BMSCs after culture for 14 d or osteogenic differentiation for 14 d. NM, normal culture medium. OM, osteogenic differentiation‐inducing medium. Scale bar = 100 μm. (C) Real‐time qPCR analysis detected mRNA expression of ALP, Collagen‐1, BMP2, BMP4, Runx2, Sp7, OCN, OPN in BMSCs cultured in NM or OM after 14 d. ALP, Alkaline phosphatase. Collagen‐1, collagen type αI. BMP2, bone morphogenetic protein 2. BMP4, bone morphogenetic protein 4. Runx2, runt‐related transcription factor. Sp7, osterix. OCN, osteocalcin. OPN, osteopontin. (D and E) The osteogenic differentiation of BMSCs exposed to different concentrations of melatonin was observed by ARS staining (D) and ALP (E) staining. Scale bar = 100 μm. (F) The osteoblast‐related genes were differentially expressed in BMSCs treated with melatonin at 1 μmol/L, 10 μmol/L or 100 μmol/L Mel. *P < 0.05, **P < 0.01 and ***P < 0.001 compared with controls

In our previous study, we found that melatonin protected BMSCs against iron overload‐induced inhibition of osteogenic differentiation, but the underlying mechanism was not characterized.^17^ In this study, we investigate the effect of melatonin on the osteogenic potential of BMSCs and its underlying mechanism. First, BMSCs were cultured in normal culture medium (NM) or osteogenic differentiation‐inducing medium (OM) for 14 days respectively. The cells were then harvested and underwent subsequent experiments. Besides, mineralization is deemed as a critical marker of osteogenesis. Then, ARS staining and alkaline phosphatase (ALP) staining were carried out to detect the mineralization nodules of BMSCs after treatment with NM or OM. ARS staining showed that OM‐treated BMSCs exhibited an increase in the number and areas of mineralization nodules, suggesting that OM treatment induced BMSCs to mature osteoblasts successfully (Figure 1A). Similarly, ALP staining confirmed that OM promoted the osteogenic differentiation in BMSCs (Figure 1B). We performed real‐time qPCR analysis to investigate whether OM affected the expression level of osteoblast‐related genes which were regarded as important genes during osteogenesis. The results revealed that mRNA expression levels of ALP, collagen type α I (Collagen‐1), bone morphogenetic protein 2 (BMP2), bone morphogenetic protein 4 (BMP4), runt‐related transcription factor (Runx2), osterix (Sp7), osteocalcin (OCN) and osteopontin (OPN) in BMSCs were all up‐regulated in the presence of OM (Figure 1C). We treated BMSCs with melatonin at a concentration of 1 μmol/L, 10 μmol/L or 100 μmol/L and then the cells were induced into osteoblasts for 14 days. Compared with undifferentiated cells, the differentiated cells showed more calcium deposition (Figure 1D,E). ARS staining revealed BMSCs treated with melatonin exhibited more calcium nodules compared with controls (Figure 1D). Moreover, among different concentrations, melatonin at 10 μmol/L promoted the greatest osteoblastic differentiation of BMSCs (Figure 1D). In addition, ALP staining indicated that the number and area of calcium nodules slightly increased after exposure to melatonin at concentration of 1‐100 μmol/L, while the marked increase in the osteogenesis of BMSCs which were treated with 10 μmol/L melatonin was detected (Figure 1E).

Real‐time qPCR analysis suggested that melatonin augmented the mRNA expression level of osteogenic‐related genes, including ALP, Collagen‐1, BMP2, BMP4, Runx2, Sp7, OCN and OPN compared with controls (Figure 1F). Importantly, 10 μmol/L melatonin‐treated BMSCs achieved a peak in the expression levels of master genes during osteogenesis (Figure 1F). Melatonin at 10 μmol/L therefore was regarded as the effective concentration and used for further analysis.

### 3.2 Beneficial effect of melatonin on the osteogenesis was blocked by luzindole

**Figure 2:**
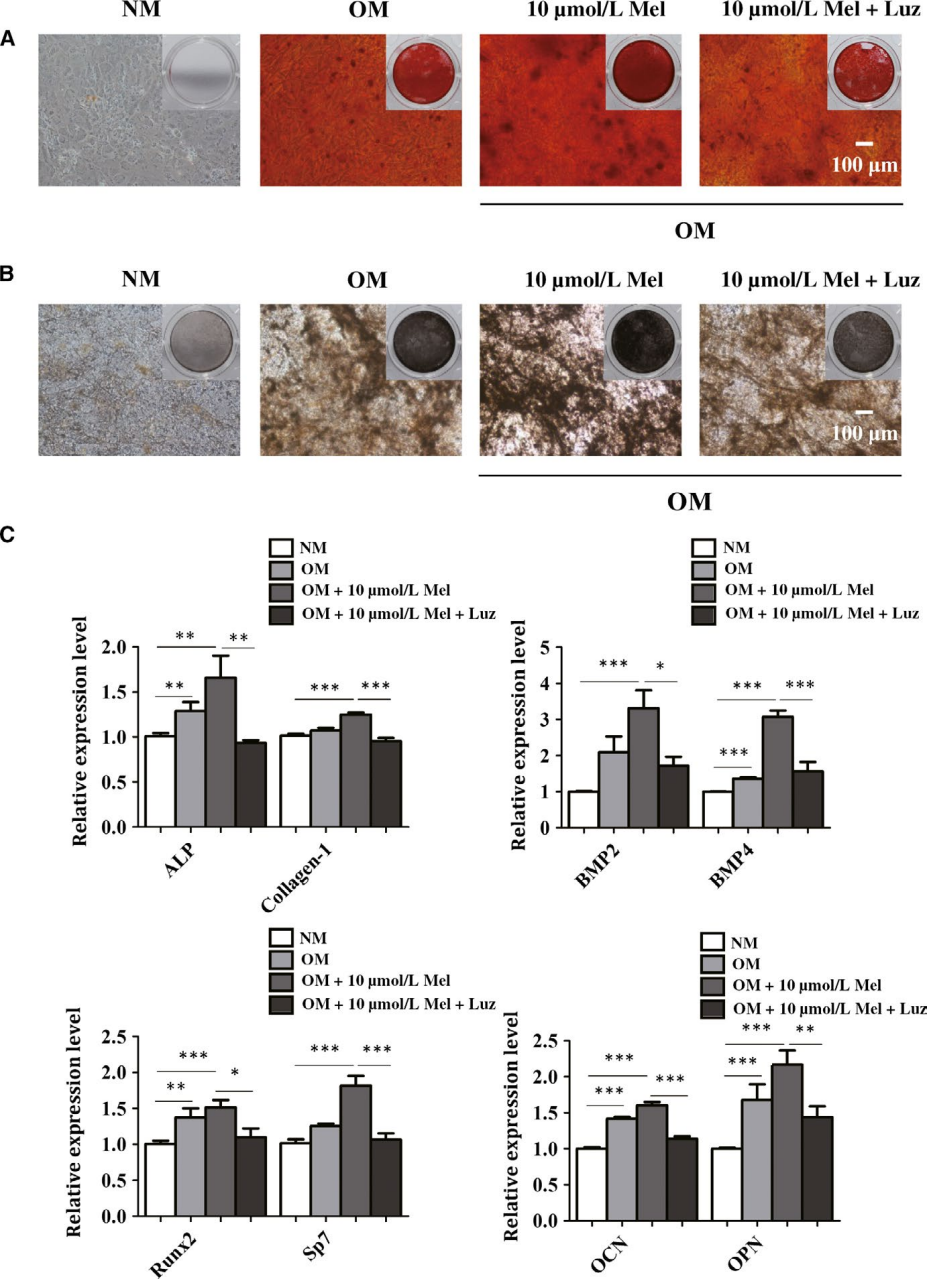
10 μmol/L melatonin promotes the osteoblast differentiation, which was abrogated by luzindole. (A) The calcium deposits of bone marrow mesenchymal stem cells (BMSCs) in the presence of normal culture medium (NM), osteogenic differentiation‐inducing medium (OM), 10 μmol/L Mel or 10 μmol/L Mel+luzindole were detected by ARS staining. Scale bar = 100 μm. Luz, luzindole. (B) ALP staining exhibited different areas and depth of colour of BMSCs in different groups after osteogenic differentiation. Scale bar = 100 μm. (C) Real‐time qPCR analysis measured the mRNA expression of osteogenic‐related genes of BMSCs after the treatment of NM, OM, 10 μmol/L or 10 μmol/L Mel Mel+luzindole. *P < 0.05, **P < 0.01 and ***P < 0.001 compared with NM‐treated group

To determine if melatonin promoted the osteogenic differentiation in a melatonin receptor‐dependent manner, we further tested the effects of luzindole (Luz), a known receptor antagonist of melatonin on melatonin‐induced osteogenic effect. We treated BMSCs with NM, OM, OM + 10 μmol/L melatonin, OM + 10 μmol/L melatonin + 10 μmol/L luzindole respectively. After differentiation of 14 days, ARS staining and ALP staining demonstrated that OM significantly enhanced the capacity of osteogenic differentiation of BMSCs, which was further promoted by 10 μmol/L melatonin treatment (Figure 2A,B). However, as shown in Figure 2A,B, the increase in the osteogenesis caused by melatonin was blocked by 10 μmol/L luzindole, suggesting melatonin receptor involvement (Figure 2A,B). In addition, our data suggested that BMSCs in the presence of 10 μmol/L melatonin exhibited a marked increase in the formation of calcium nodules, which sharply declined in luzindole‐treated cells (Figure 2A,B). Moreover, the obtained results uncovered that the expression level of markers for osteoblast was obviously increased by melatonin at a concentration of 10 μmol/L compared with control group, which was visibly reduced in the presence of luzindole (Figure 2C). Thus, the osteoblast formation potential of BMSCs caused by melatonin was blocked by luzindole, suggesting that the melatonin receptor was involved in the process.

### 3.3 MiR‐92b‐5p promotes the differentiation of BMSCs into osteoblasts

**Figure 3:**
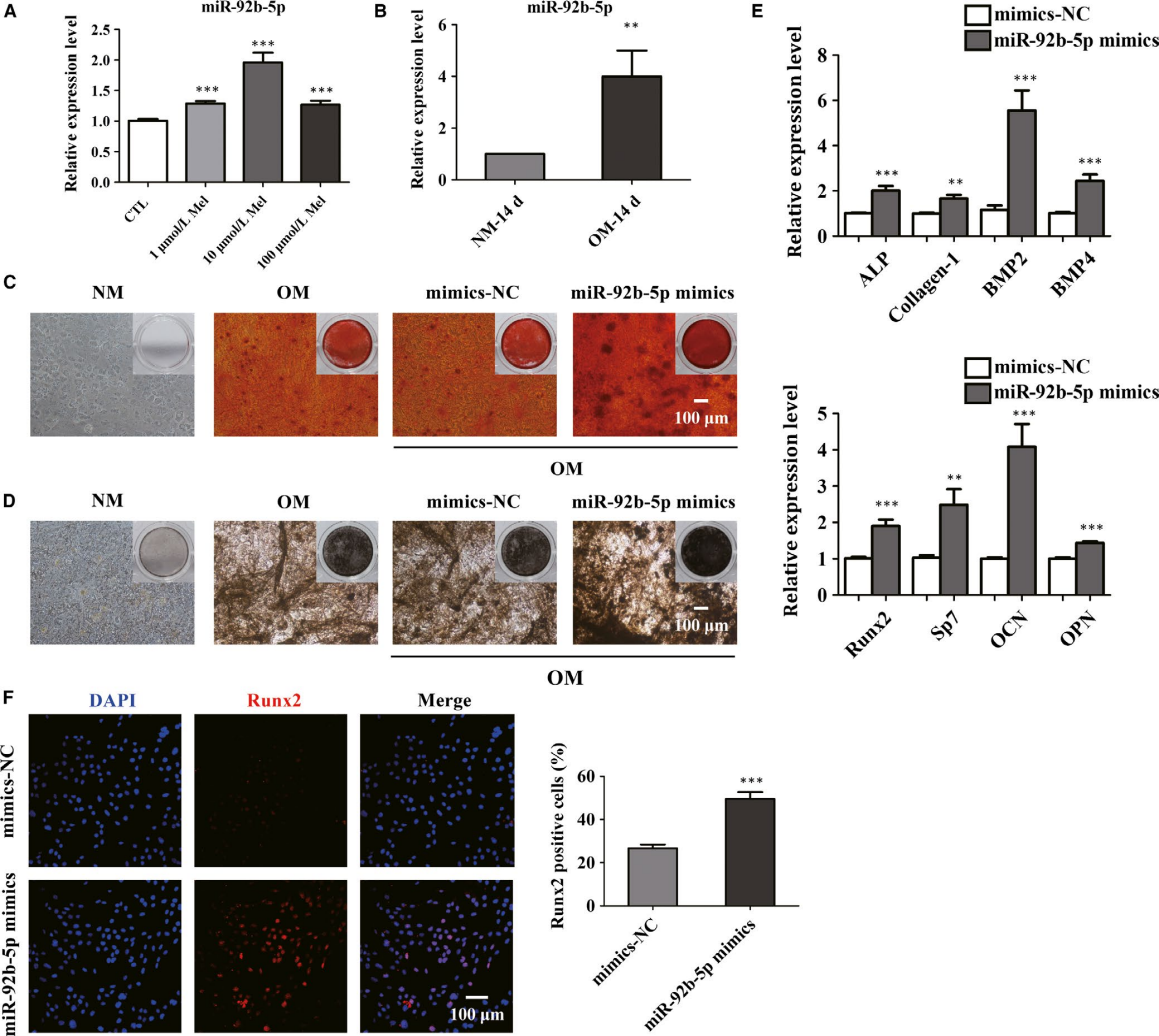
The effect of miR‐92b‐5p on the osteoblastic differentiation of bone marrow mesenchymal stem cells (BMSCs). (A) The expression of miR‐92b‐5p in BMSCs after treatment with various concentrations of melatonin was measured. (B) Real‐time qPCR technique detected the expression of miR‐92b‐5p between normal culture medium (NM)‐treated BMSCs and OM‐treated BMSCs for 14 d. (C and D) ARS staining (C) and ALP (D) staining investigated the ability of osteogenesis in BMSCs transfected with miR‐92b‐5p mimics. Scale bar = 100 μm. (E) MiR‐92b‐5p mimics elevated the mRNA expression level of osteoblast markers after induction for 14 d. NC, negative control. (F) Immunofluorescence staining visualized the percentage of Runx2‐positive cells in the presence of miR‐92b‐5p mimics or mimics‐NC. Scale bar = 100 μm. **P < 0.01 and ***P < 0.001 compared with mimics‐NC

Given the fact that melatonin accelerated the osteoblast differentiation of BMSCs, we have been suggested that the function might be in connection with the expression of miRNAs. To further investigate the mechanism of the effect of melatonin on BMSCs, real‐time qPCR was performed to examine the expression level of miRNAs in BMSCs treated with different concentrations of melatonin. Real‐time qPCR analysis revealed that the expression of miR‐92b‐5p began to increase after melatonin administration and peaked at 10 μmol/L melatonin (Figure 3A). We then examined the expression level of miR‐92b‐5p in BMSCs after osteoblast differentiation. From the results of real‐time qPCR, far higher expression of miR‐92b‐5p was observed in BMSCs after osteogenic induction of 14 days (Figure 3B). Therefore, we supposed that melatonin might facilitate osteoblast differentiation by elevating the expression of miR‐92b‐5p. Accordingly, from the results of ARS staining, it was noted that cells transfected with miR‐92b‐5p mimics exhibited more calcium deposits and increased number of osteoblasts, which further proved that miR‐92b‐5p stimulated osteogenesis of BMSCs (Figure 3C). Similarly, ALP staining confirmed that transfection of miR‐92b‐5p effectively enhanced the ability of osteogenesis in BMSCs compared with negative control (Figure 3D). For further validation, the expression level of osteoblast marker genes was analysed by real‐time qPCR. Intriguingly, real‐time qPCR showed that cells pre‐treated with miR‐92b‐5p mimics displayed higher expression level of ALP, Collagen‐1, BMP2, BMP4, Runx2, Sp7, OCN and OPN in comparison with its negative control (Figure 3E). Also, immunofluorescent staining revealed that the stimulatory effect of miR‐92b‐5p mimics on osteoblast differentiation was consistent with the results above (Figure 3F). Therefore, the data suggested that miR‐92b‐5p promoted the differentiation of BMSCs into mature osteoblasts.

### 3.4 Silencing of miR‐92b‐5p inhibits the osteogenesis of BMSCs

**Figure 4:**
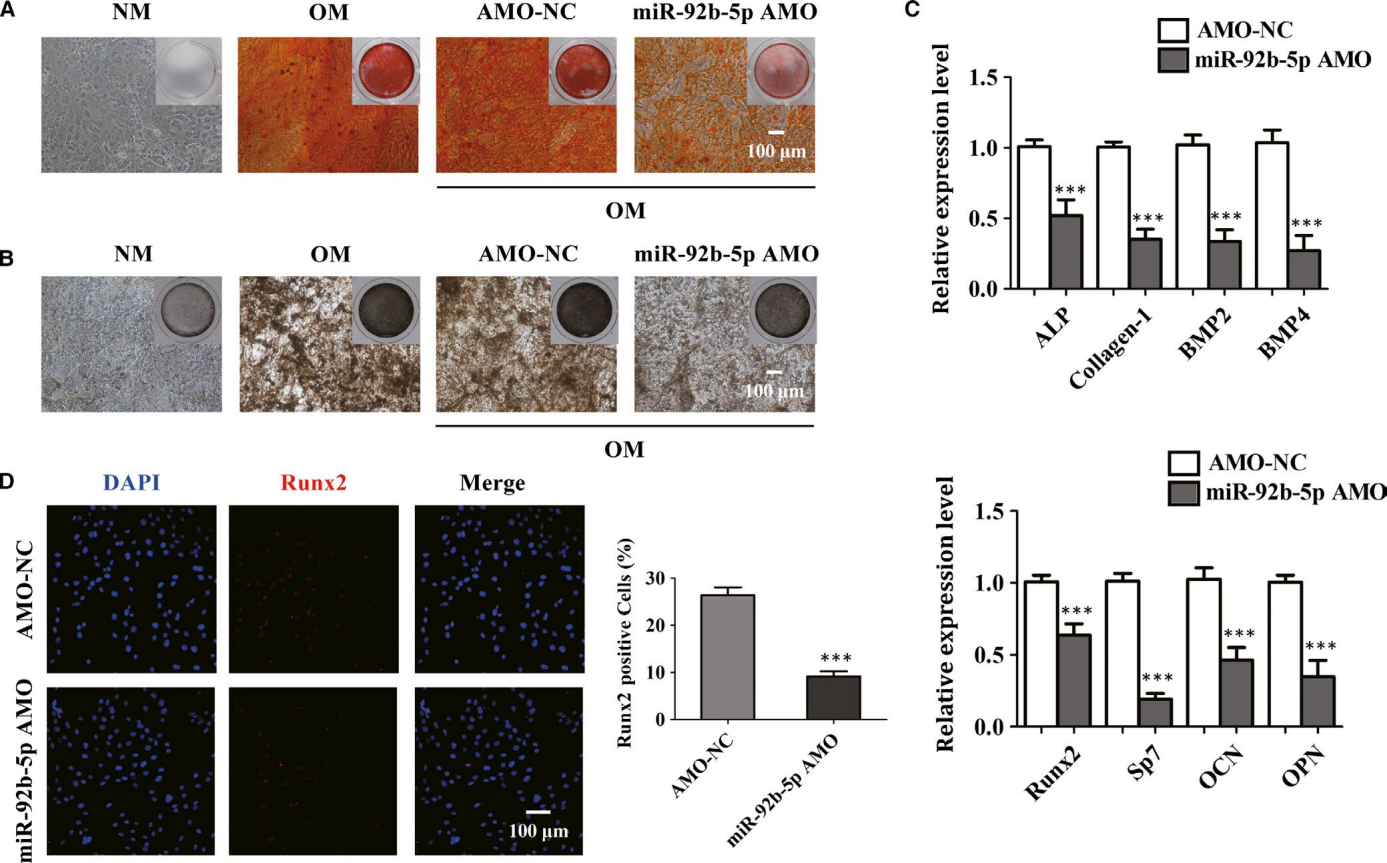
MiR‐92b‐5p AMO inhibits osteogenesis in bone marrow mesenchymal stem cells (BMSCs). (A and B) ARS (A) staining showed that miR‐92b‐5p AMO decreased the mineralization in BMSCs, which was in accordance with the result of alkaline phosphatase (ALP) staining (B). Scale bar = 100 μm. (C) The expression levels of ALP, Collagen‐1, BMP2, BMP4, Runx2, Sp7, OCN and OPN were determined by real‐time qPCR analysis. (D) Immunofluorescence staining visualized the percentage of Runx2‐positive cells. Scale bar = 100 μm. *P < 0.05, **P < 0.01 and ***P < 0.001 compared with BMSCs transfected with AMO‐NC

It was interesting to note that miR‐92b‐5p played a vital role in the differentiation of BMSCs into osteoblasts. Thus, we further investigated the role of miR‐92b‐5p AMO in the committed differentiation of BMSCs. MiR‐92b‐5p AMO and its NC were transfected into BMSCs respectively. As expected, BMSCs transfected with miR‐92b‐5p AMO exhibited fewer calcium nodules and a reduced number of osteoblasts in contrast with NC after osteogenic differentiation of 14 days (Figure 4A,B). Consistently, the decline in the expression levels of osteogenic‐related genes in BMSCs after exposure to miR‐92b‐5p AMO was quantified by real‐time qPCR analysis (Figure 4C). Immunofluorescence staining also revealed that knockdown of miR‐92b‐5p obviously reduced the expression of Runx2, a vital transcription factor during osteogenesis (Figure 4D). Together, the above results showed that miR‐92b‐5p AMO treatment led to decreased osteoblast differentiation of BMSCs.

### 3.5 Osteoblast differentiation elevated by melatonin was abrogated by miR‐92b‐5p AMO

**Figure 5:**
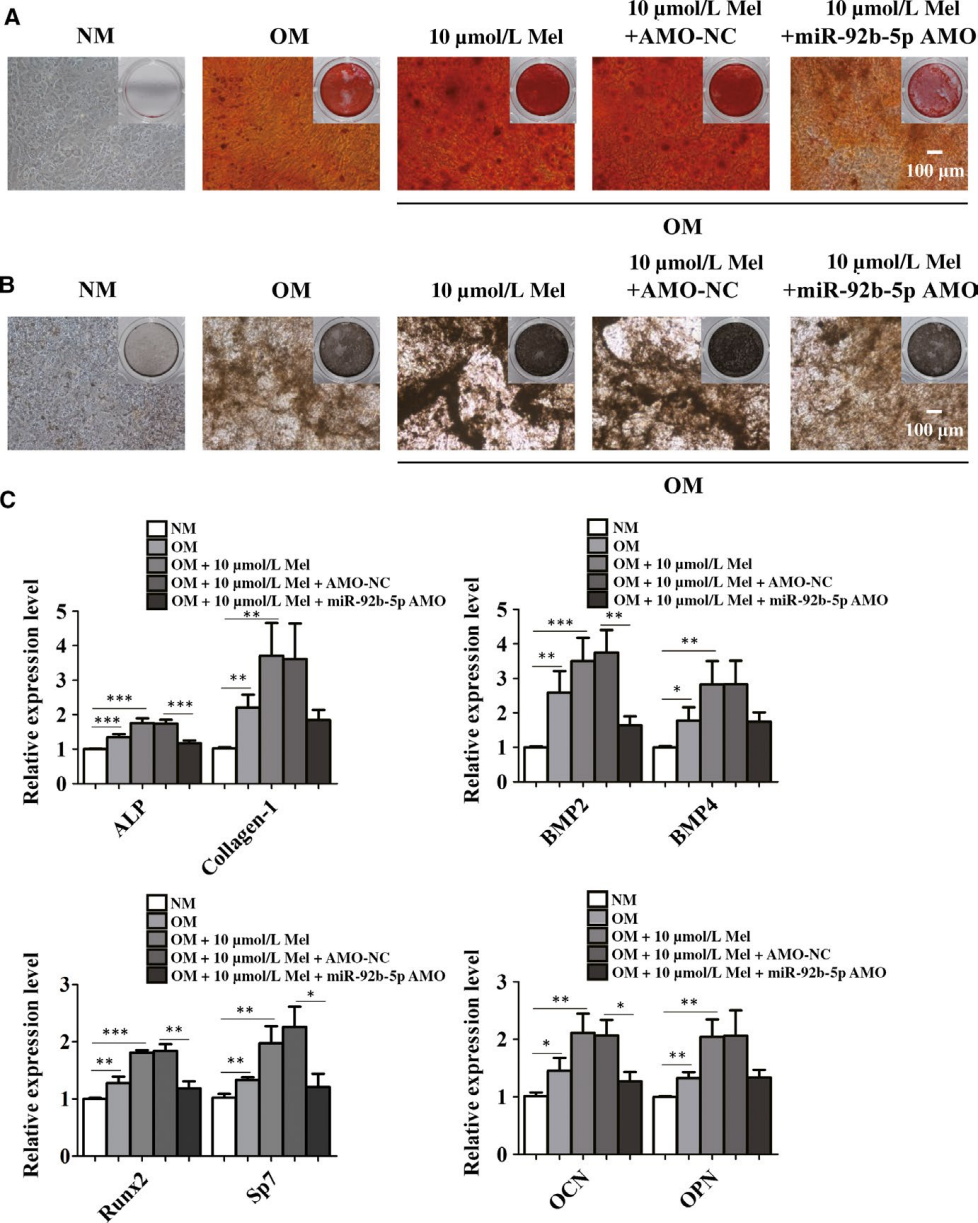
MiR‐92b‐5p AMO reversed melatonin‐induced promotion of the differentiation into osteoblasts. (A and B) The increased in osteogenesis by 10 μmol/L Mel was inhibited by miR‐92b‐5p AMO transfection. Scale bar = 100 μm. (C) The expression of osteoblast marker genes was up‐regulated by melatonin treatment, but reversed by the knockdown of AMO. *P < 0.05, **P < 0.01 and ***P < 0.001 compared with NC

To clarify the mechanisms as to how melatonin regulated osteogenesis by increasing the expression of miR‐92b‐5p, we treated BMSCs with 10 μmol/L melatonin and miR‐92b‐5p AMO. After a 14‐day osteogenic induction, the size and intensity of calcium deposition were markedly higher in the OM‐induced group than that in the NM‐treated group (Figure 5A,B). The osteoblast differentiation was increased after treatment with 10 μmol/L melatonin, while knockdown of miR‐92b‐5p delayed the osteogenesis as revealed by ARS staining (Figure 5A). Meanwhile, we found that miR‐92b‐5p AMO resulted in decreased number and area of mineralized nodules which were elevated by 10 μmol/L melatonin as observed in ALP staining (Figure 5B). According to real‐time qPCR analysis, inhibition of miR‐92b‐5p led to the down‐regulation of osteoblast‐related genes, which were significantly up‐regulated by treatment with 10 μmol/L melatonin (Figure 5C). These results clearly documented that melatonin accelerated osteoblast differentiation, which was reversed by miR‐92b‐5p AMO in BMSCs. These results suggested that a close association existed between the expression of miR‐92b‐5p and the role of melatonin in the osteogenesis of BMSCs. Thus, the above data confirmed that melatonin regulated the osteogenesis through enhancing the expression of miR‐92b‐5p.

### 3.6 miR‐92b‐5p restored osteogenesis of BMSCs from OVX‐induced osteoporotic mice

**Figure 6:**
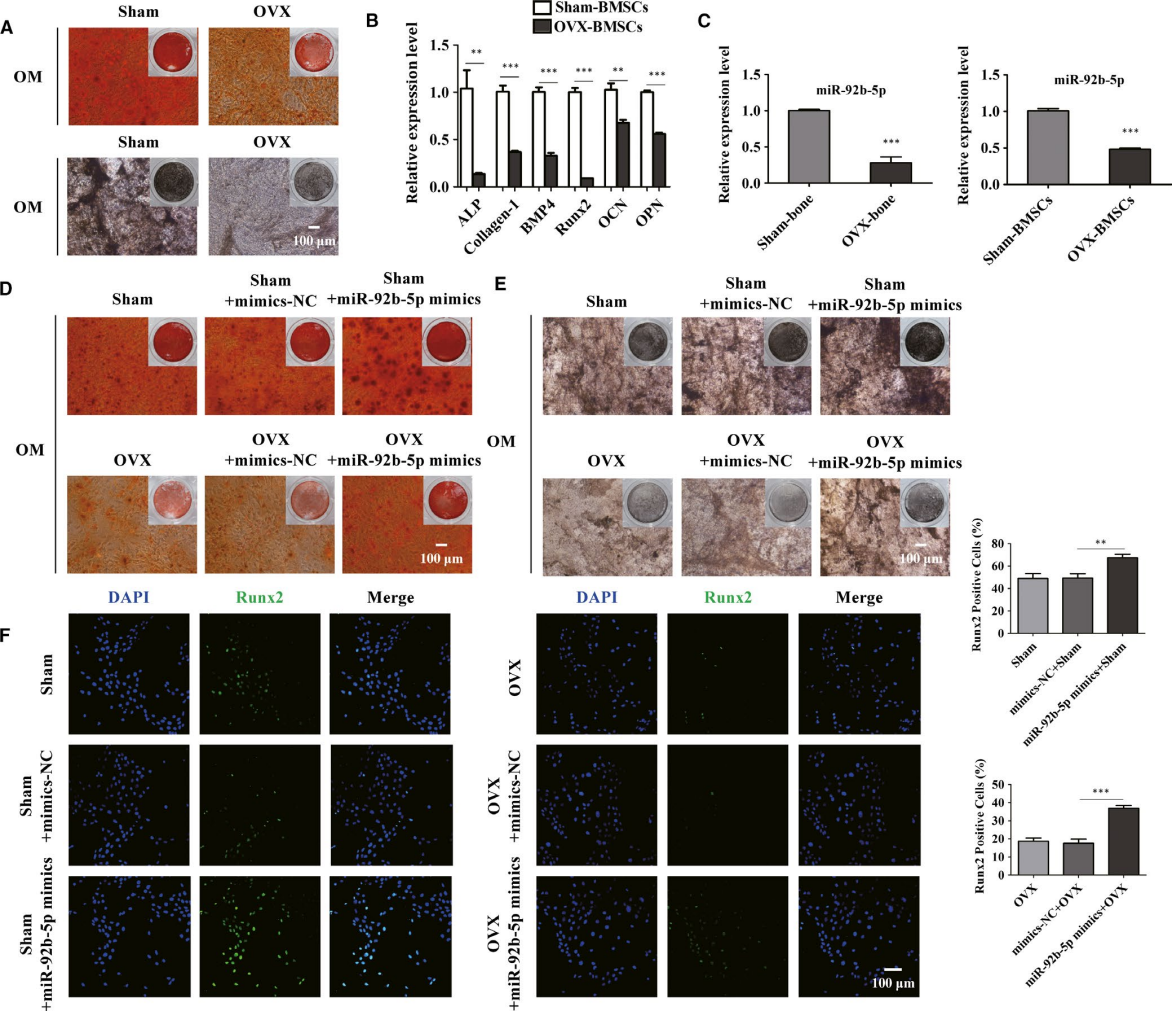
MiR‐92b‐5p reduces the osteoporosis‐induced dysfunction of osteogenesis in bone marrow mesenchymal stem cells (BMSCs). (A) Osteogenic ability of BMSCs of OVX‐induced osteoporotic mice was analysed by alizarin red S (ARS) and alkaline phosphatase (ALP) staining. Scale bar = 100 μm. (B) Real‐time qPCR was used to examine the expression of osteoblast‐related genes. (C) The expression of miR‐92b‐5p in bone tissues (left) and BMSCs (right) from Sham and OVX group. (D and E) BMSCs from Sham and OVX group were cultured in osteogenic differentiation‐inducing medium (OM) and subjected to ARS staining (left) and ALP (right) staining. Scale bar = 100 μm. (F) The percentage of Runx2‐positive cells in total BMSCs isolated from Sham and OVX mice was calculated. Scale bar = 100 μm. **P < 0.01 and ***P < 0.001 compared with cells treated with negative control

To detect the role of miR‐92b‐5p in BMSCs from osteoporotic mice, we established Sham and OVX‐induced osteoporotic mice. The osteoporotic models were successfully conducted (Figure 6A,B). Based on the above results, we further determined the role of miR‐92b‐5p in the osteoblast differentiation of BMSCs isolated from OVX‐induced osteoporotic mice. First, we identified that miR‐92b‐5p was differentially expressed in mice between Sham group and OVX group. The expression of miR‐92b‐5p was distinctly decreased in BMSCs and bone tissues from osteoporotic mice (Figure 6C). Therefore, we supposed that miR‐92b‐5p might be involved in the development of osteoporosis. ARS staining and ALP staining were performed to assess the effect of miR‐92b‐5p on the potential of osteogenic differentiation in BMSCs of mice from the Sham and OVX groups. ARS staining indicated that BMSCs from osteoporotic mice displayed fewer calcium deposits than the control group (Figure 6D). However, the decrease in the osteogenic differentiation caused by OVX surgery was elevated by miR‐92b‐5p transfection (Figure 6D). Moreover, as shown in ALP staining, osteogenesis was thoroughly impaired in OVX‐induced osteoporotic mice compared with Sham group (Figure 6E). However, the sharp decline in the osteogenic differentiation of BMSCs from OVX mice was restored by miR‐92b‐5p mimics (Figure 6E). Comparisons in the protein expression of Runx2 between these two groups were conducted using immunofluorescence analysis. As shown, the number of the Runx2‐positive BMSCs was visibly decreased in the OVX‐induced group compared with Sham group (Figure 6F). In addition, the percentage of Runx2‐positive cells was elevated by miR‐92b‐5p transfection (Figure 6F). These results revealed that miR‐92b‐5p restored the attenuation in osteogenesis of BMSCs which was caused by osteoporosis, and the therapeutic increase in miR‐92b‐5p in BMSCs counteracted the impaired osteogenesis in the osteoporotic models.

### 3.7 miR‐92b‐5p was involved in the osteogenesis by directly targeting ICAM‐1

**Figure 7:**
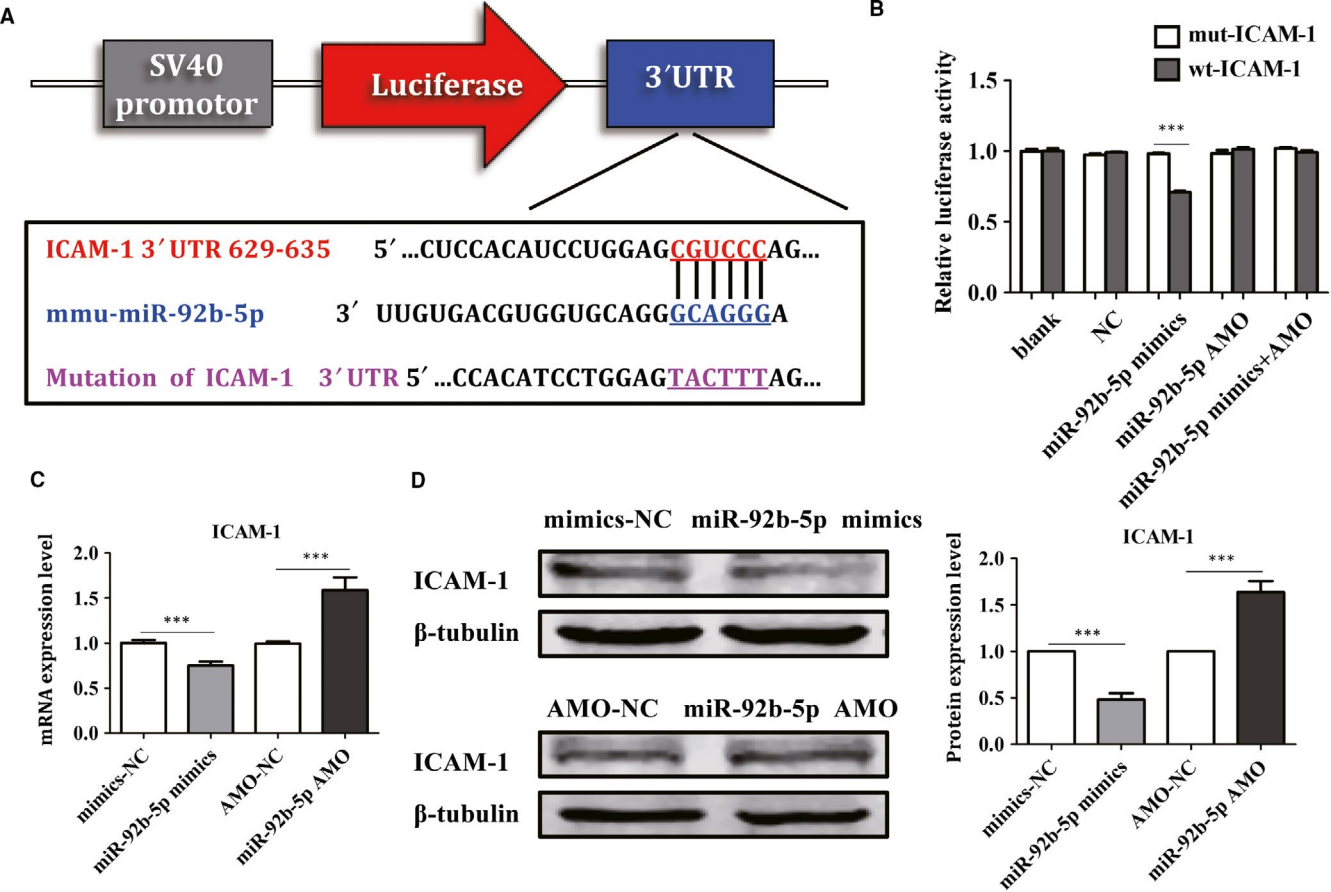
MiR‐92b‐5p regulates osteogenesis by targeting ICAM‐1. (A) The binding sequence of miR‐92b‐5p and ICAM‐1. (B) The luciferase reporter assay was used to analyse the regulation of miR‐92b‐5p and ICAM‐1. (C) Real‐time qPCR analysis indicated miR‐92b‐5p regulated the expression of ICAM‐1. (D) Western blot was performed to detect the protein expression of ICAM‐1 after transfection of miR‐92b‐5p. ***P < 0.001 compared with cells in the presence of NC

To explore the underlying mechanism related to the function of miR‐92b‐5p in the osteogenesis of BMSCs, we predicted the possible target genes of miR‐92b‐5p by TargetScan online. ICAM‐1 was suggested as the candidate gene for miR‐92b‐5p because of the high‐potential binding sites between miR‐92b‐5p and ICAM‐1 (Figure 7A). Meanwhile, it has been uncovered that ICAM‐1 plays a critical role in osteogenesis. As shown in Figure 7B, the luciferase reporter assay revealed that the relative luciferase activity was down‐regulated in cells treated with miR‐92b‐5p mimics and ICAM‐1, while the association was completely abrogated when the 3′‐UTR of ICAM‐1 was mutated. Furthermore, real‐time qPCR and Western blot analysis documented that miR‐92b‐5p mimics effectively reduced the expression levels of ICAM‐1, while miR‐92b‐5p AMO elevated the expression of ICAM‐1 (Figure 7C,D). The above results suggested that miR‐92b‐5p participated in the osteogenesis of BMSCs by directly targeting ICAM‐1.

## 4 DISCUSSION

In this study, we revealed that melatonin regulates the osteogenic differentiation through a melatonin receptor pathway. Furthermore, we found that melatonin accelerates the osteogenesis by improving the expression of miR‐92b‐5p and, miR‐92b‐5p enhances the differentiation into mature osteoblasts by targeting ICAM‐1. This study provides more insights into the role and mechanism of miRNAs in regulating osteogenesis of BMSCs.

Osteoporosis is a severe progressive systemic skeletal disease which causes humpback, osteodynia, thoracocyllosis and especially fragility fractures.^37^ Osteoporosis is caused by the imbalance between osteoclast‐mediated bone resorption and osteoblast‐induced bone formation of osseous tissues. The present attempts to treat osteoporosis have focused on exercising, calcium supplement and vitamin D administration.^38^ Besides these existing treatment methods, new strategies to effectively prevent the development of osteoporosis by improving bone formation are always urgently required.

Melatonin is a major indoleamine produced and secreted by pineal gland; melatonin exhibits a variety of biological actions.^39^, ^40^ Recently, melatonin has been reported to participate in the differentiation of BMSCs.^41^, ^42^ For example, it has been demonstrated that melatonin dose‐dependently activated osteogenesis and wound healing of pre‐osteoblastic MC3T3‐E1 cells via the BMP/ERK/Wnt pathways.^43^ In another study, melatonin restored TNFα‐induced decrease in the osteogenesis of human mesenchymal stem cells (hMSCs) by regulating bone morphogenetic protein‐SMAD1 signalling bone morphogenetic protein‐SMAD1 signalling. In addition, previous study reported that melatonin treatment directly inhibited adipogenesis and simultaneously accelerated osteogenesis of hMSCs in a dose‐dependent manner and it affected the osteogenesis and adipogenesis by enhancing Runx2 expression and by suppressing PPARγ expression.^10^ Similarly, our study revealed that melatonin may promote the osteogenesis of BMSCs. Many have demonstrated that melatonin exerted its biological actions via MT‐dependent or independent manner. In the present study, we found that melatonin induced the osteoblast differentiation of BMSCs in a MT‐dependent manner, which is in agreement with previous reports.^17^

MicroRNAs have been shown to play important roles in the occurrence and development of osteoporosis. Several miRNAs such as miR‐675 in cardiac progenitor cells, miR‐16‐5p in gastric cancer cells, miR‐34a in neurons were proved to be regulated by melatonin treatment.^27^, ^35^, ^44^ There are, however, no reports demonstrating an association between melatonin‐induced osteogenesis and miRNAs as an underlying mechanism. Therefore, we further tested whether melatonin regulates osteoblastic differentiation of BMSCs by modulating the expression of miRNAs. In the present study, we treated BMSCs with different concentrations of melatonin and found that 10 μmol/L was the most effective concentration for enhancing the capacity of osteoblast differentiation of BMSCs. We found that the expression of miR‐92b‐5p was significantly increased after treatment with 10 μmol/L melatonin. Previous studies have showed that miR‐92b‐5p was related to acute heart failure, infant respiratory virus infection, coronary microembolization and early‐onset atrial fibrillation, but there are no reports related to the role of miR‐92b‐5p in the BMSC fate.^45^, ^46^, ^47^ Our data showed that miR‐92b‐5p facilitates osteoblast differentiation of BMSCs and reverses the osteoporosis‐induced impaired osteogenic potential of BMSCs isolated from OVX‐induced mice. Further investigation revealed that miR‐92b‐5p exhibited a positive role in the osteogenic differentiation of BMSCs via modulating ICAM‐1 which been demonstrated to play a role in the osteogenic differentiation and bone regeneration in the previous studies.^48^ ICAM‐1 has been reported to inhibit the osteogenesis of BMSCs and serve as a new molecular target to accelerate bone regeneration and repair in inflammatory microenvironments.^49^

According to a randomized controlled trial, melatonin treatment increases BMD and integrity of the femoral neck in post‐menopausal women with osteopenia.^50^ In that report, 1‐3 mg melatonin per day increases BMD of femoral neck in a dose‐dependent manner compared with placebo treatment, as BMD increased by 0.5% in the 1 mg/d group and by 2.3% in the 3 mg/d group. Besides, it has been reported that trabecular thickness in tibia was improved by 2.2% and BMD in the spine was increased by 3.6% after 3 mg/d melatonin treatment. Consistently, our study discovered that 1 μmol/L, 10 μmol/L or 100 μmol/L melatonin significantly promoted the osteogenic differentiation of BMSCs and reversed the impaired potential of osteogenesis in OVX‐related osteoporotic mice.

Similar to the reports, melatonin accelerates the differentiation of BMSCs into osteoblast in a concentration‐dependent manner; and melatonin treatment makes it a hopeful method in the future prevention of bone loss and fracture. Both studies proposed that melatonin is a potential treatment for improving human bone balance and affecting bone health.

To the best of our knowledge, the present study is the first to provide evidence about the correlation between melatonin and miRNAs during osteoblast differentiation in BMSCs. Melatonin may regulate osteoblastic differentiation of BMSCs in a melatonin receptor‐dependent manner. Furthermore, our findings suggest that miR‐92b‐5p restores the osteoporosis‐induced decline in the osteogenic potential of BMSCs. This study helps to clarify the effects of melatonin in the osteogenic differentiation and its mechanism. These findings open up new possibilities for melatonin use as an effective strategy to maintain bone metabolism in patients with osteoporosis.

In conclusion, we found that melatonin stimulates osteogenesis of BMSCs by increasing the expression of miR‐92b‐5p which directly targets at ICAM‐1. Thus, the present study suggests a possible application of melatonin as a therapeutic agent for osteoporosis.

## CONFLICTS OF INTEREST

The authors indicate no potential conflicts of interest.

## AUTHORS CONTRIBUTION

L Yang, BZ.Cai and F Yang designed the experiments and wrote the manuscript. Y Li, C Feng and MQ Gao carried out the experiments. MY Jin and TY Liu performed real‐time qPCR and Western blot analysis. Y Yuan, GG Yan, R Gong, Y Sun, MY He and YT Fu provided the technical support. L Zhang, Q Huang, FZ Ding, WY Ma, ZG Bi and CQ Xu contributed to the data collection. N Sukhareva, D Bamba and R Reiters performed the data analysis and revised the manuscript. All the authors read and approved the final manuscript.
